# Ultrasonic-microwave-assisted extraction for enhancing antioxidant activity of *Dictyophora indusiata* polysaccharides: The difference mechanisms between single and combined assisted extraction

**DOI:** 10.1016/j.ultsonch.2023.106356

**Published:** 2023-03-06

**Authors:** Yanlin Zhang, Yi Lei, Shirong Qi, Mingxuan Fan, Shuyi Zheng, Qingbin Huang, Xu Lu

**Affiliations:** a College of Food Science, Fujian Agriculture and Forestry University, 15 Shangxiadian Road, 350002 Fuzhou, China; b Teagasc Food Research Centre, Food Chemistry and Technology Department, Moorepark, Fermoy, Co. Cork, Ireland; c Dendrobium Candidum Science and Technology Institute of Liancheng County, 1-2 Xiewu Road in Luwu Village Jiele Township, 366200 Longyan, China; d China-Ireland International Cooperation Center for Food Material Science and Structure Design, Fujian Agriculture and Forestry University, 350002 Fuzhou, China

**Keywords:** *Dictyophora indusiata*, Polysaccharides, Extraction, Response surface, Structures, Antioxidant

## Abstract

The purpose of this study is to investigate the effects of different extraction methods (hot water-assisted extraction (HWE), microwave-assisted extraction (MAE), ultrasonic-assisted extraction (UAE), and ultrasonic-microwave- assisted extraction (UAME)) on the yield, chemical structures and antioxidant activity of *Dictyophora indusiata* polysaccharides (DPs). The research results showed that UMAE treatment had greater degree of damage to the cell wall of DPs and better comprehensive antioxidant capacity. Different extraction methods had no obvious effect on the types of glycosidic bonds and sugar rings, similar chemical composition and monosaccharide composition, with different absolute molecular weight (*Mw*) and molecular conformation. In particular, DPs for UMAE method had the highest polysaccharides yield, which was related to the conformational stretching and degradation avoidance of DPs in the higher molecular weight components under the simultaneous action of microwave and ultrasonic. These findings suggest that the UMAE technology has good potential for modification and application of DPs in the functional food industry.

## Highlights

• The optimal extraction process of polysaccharides by microwave ultrasonic was determined.
• No superposition effect under both microwave and ultrasound methods alone or in combination for polysaccharides.
• The polysaccharides extracted by the four methods have different interaction patterns with water.
• Fractions in polysaccharides have effect on the antioxidant activity of extraction product.
• Relationships between extraction methods, nutrient content, structures and antioxidant activity were established.

## Graphical abstract

**Figure:**
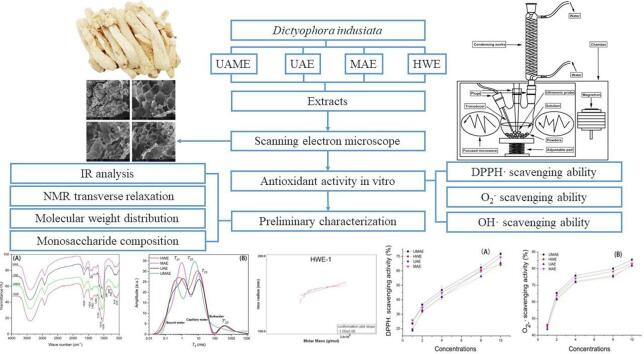

## 1 Introduction

With the continuous development of mushroom cultivation methods, many mushrooms have been cultivated on a commercial scale as food and medicinal ingredients. Among them, *Dictyophora indusiata* is one of the edible and medicinal mushrooms, usually distributed in Asian regions, due to its beauty known as the “Queen of Mushrooms” for appearance, unique taste and health benefits [12]. A variety of biologically active substances have been discovered from *Dictyophora indusiata* in recent years, such as proteins, polysaccharides, vitamins, etc. *Dictyophora indusiata* polysaccharides also have immunomodulatory, anti-inflammatory and anti-tumor activities [33], which is related to the antioxidant activity of polysaccharides [31]. However, there is still a lack of in-depth research on the reasons for the differences in the in vitro antioxidant activity of polysaccharides, which limits its application in a wider range.

Polysaccharides are polar macromolecular compounds, and the extraction yield, quality, chemical structure and biological activity depend on the factors such as extraction methods and plant cell walls [4], [11]. In recent years, acid extraction, alkaline extraction, enzyme-assisted extraction, ultrasonic-assisted extraction and microwave-assisted extraction are often used to improve the extraction efficiency of polysaccharides [28]. However, solvents extraction has been shown to have any destructive effects on sugar residue of polysaccharides, which further affect antioxidant properties [34], [40]. In the process of ultrasonic-assisted extraction of fungal polysaccharides, the cavitation bubbles generated by ultrasonic waves caused tissue rupture and thus accelerate mass transfer [34]. It has been found that the extracted *Ganoderma lucidum β*-D-glucan has higher molecular weight and optimal branching degree, which has better antioxidant activity *in vitro* [2], [7]. Microwave-assisted extraction greatly reduces solvent usage and shortens preparation time, which radiation breaks down the cell walls, allowing the contents to better come into contact with the solvent [42]. However, it was also found that the antioxidant activity of polysaccharides extracted by ultrasonic was worse than that by microwave treatment [28]. Ultrasound/microwave-assisted extraction (UMAE) is a complementary technology has emerged in recent years, which realizes rapid, uniform and low-temperature extraction, and overcomes the shortcomings of both technology [29], [37], Srivastava et al. [27]. So far, there are few reports on the in-depth study of the relationship between the polysaccharide components, structure conformation and antioxidant activity of *Dictyophora indusiata* polysaccharides by different extraction methods, especially the combination of microwave and ultrasonic extraction methods.

Therefore, the optimal process of ultrasonic-microwave-assisted extraction of *Dictyophora indusiata* polysaccharides was first optimized, and then compared with the nutritional components and antioxidant activities of *Dictyophora indusiata* water-soluble polysaccharides extracted by hot water, ultrasonic, and microwave. The components, groups and conformational changes of polysaccharides were studied using scanning electron microscopy, fourier transform infrared spectroscopy, multi-angle laser scattering and low-field nuclear magnetic resonance techniques. It is beneficial to clarify the antioxidant activity of polysaccharides during the extraction process and mechanism of the difference, which could provide assistance for further development of functional polysaccharides application.

## 2 Materials and methods

### 2.1 Materials

*Dictyophora indusiata* was purchased in Nanping Shengli Market. After the *Dictyophora indusiata* is air-dried, it is fully ground into powder by a high-speed pulverizer. DPPH (1,1-diphenyl-2-picroyl), ABTS (total antioxidant activity kit), PMS, NATB were purchased from Sigma Company in the United States (America). EDTA, trichloroacetic acid, ascorbic acid, Tris-hydrochloric acid buffer, H_2_O_2_ absolute ethanol, chloroform, sodium chloride, concentrated sulfuric acid, chloroform, *n*-butanol were purchased from Shanghai Chemical Reagent Factory (Shanghai, China). Methanol, trifluoroacetic acid, sodium hydroxide and sodium acetate trihydrate were purchased from sigma (Sigma–Aldrich, St. Louis, MO, USA). The chemicals and solvents used in this study were of analytical grade.

### 2.2 Extraction of *Dictyophora indusiata* polysaccharides by various methods

#### 2.2.1 Hot water extraction

5 g of dried *Dictyophora indusiata* fruiting body powder was taken with 200 mL of deionized water at a 1:40 material-to-liquid ratio, and moved in a warm water bath at 88 °C (Tianjin Aotesiens Instrument Co., Ltd., Tianjin, China) to extract 2.5 h. Then, the supernatant was separated by centrifuging (5000 r/min, 10 min) after cooling to room temperature. The residue was extracted twice in the same way and combined with supernatant. According to the steps described, the *DPs* were separated, preliminarily purified and freeze-dried.

#### 2.2.2 Ultrasonic assisted hot water extraction

5 g of dried *Dictyophora indusiata* fruiting body powder was taken with 200 mL of deionized water at a solid–liquid ratio of 1:40, and moved in an ultrasonic-microwave combined reaction system (XH-300B, Beijing Xianghu Technology Development Co., Ltd., Beijing, China). The extraction was performed at a constant temperature of 600 W at 80 °C for 25 min. After the reaction was terminated, the supernatant was separated, the residue was extracted twice in the same manner, and the supernatants were combined. The *DPs* were separated, preliminarily purified and freeze-dried.

#### 2.2.3 Microwave-assisted hot water extraction

5 g of dried *Dictyophora indusiata* fruiting body powder was taken with 200 mL of deionized water at a solid–liquid ratio of 1:40, and moved in an ultrasonic-microwave combined reaction system (XH-300B, Beijing Xianghu Technology Development Co., Ltd., Beijing, China). Among them, extraction was performed for 7 min under the conditions of zero ultrasonic power and 150 W of microwave power. After the reaction was terminated, the supernatant was separated. The residue was extracted twice in the same manner and combined with supernatants. The *DPs* were separated, preliminarily purified and freeze-dried.

#### 2.2.4 Combined microwave-ultrasonic method

The fruiting bodies of *Dictyophora indusiata* after being crushed by a plant crusher (FZ102, Tianjin Test Instrument Co., Ltd., Tianjin, China) were passed through a 60-mesh sieve and used for later use. The sieved *Dictyophora indusiata* powder was added into distilled water (self-provided) according to the fixed material-to-liquid ratio, and extracted under different microwave-ultrasonic (UMAE) wave conditions. The extracted feed liquid was cooled to room temperature and centrifuged (4000 × g, 8 min). The supernatant was separated, the precipitate was extracted twice under the same conditions, and the supernatants were combined. Evaporate (50 °C) to 1/4 of the combined volume with a rotary evaporator (RE-52A, Yarong Biochemical Instrument Factory, Shanghai, China), then add the concentrate to 4 times volume of anhydrous ethanol at 4 °C After standing for 12 h, centrifuge (4500 × g, 8 min). Finally the separated precipitate was washed with absolute ethanol and freeze-dried to obtain the crude polysaccharide. Wherein the content of polysaccharide adopts phenol–sulfuric acid method to measure, is calculated according to the following formula:

(1) $$\text{Polysaccharides yield}\left(\%\right)=\frac{\text{Polysaccharides quality}(mg)}{\text{Sample quality}(\text{mg})}\times 100\%$$

### 2.3 Response surface optimization of ultrasonic-microwave combined extraction of polysaccharides experimental design

Taking the yield of polysaccharides (%) as the investigation index, the effects of extraction time (min), ultrasonic power (W), microwave power (W), and liquid-to-material ratio (mL/g) on the yield of polysaccharides were studied. On the basis of the single factor test results, taking the yield of polysaccharides as the response value, the center combination test (CCD) method was used to carry out a four-factor and three-level experimental design, and the extraction time (*X_1_*), ultrasonic power (*X_2_*), microwave power were determined. The effect of (*X_3_*) and liquid-material ratio (*X_4_*) on the yield of polysaccharides; the response surface includes 30 test points, 6 of which are central points, and the regression equation is obtained from the response surface to optimize the best process parameters of polysaccharides.

### 2.4 Analysis of physicochemical properties of *Dictyophora indusiata* after extraction (scanning electron microscope)

The structural features of *Dictyophora indusiata* after extraction were determined by Fourier transform infrared spectrophotometer (Perkin Elmer Corp., USA). 1 mg of sample was mixed with 100 mg of dry KBr and compressed into 1 mm pellets for analysis. Infrared spectra were recorded in the 400–4000 cm^−1^ range on fourier transform infrared spectrophotometer (VECTOR-22).

### 2.5 Determination of antioxidant activity in vitro

Take 100 mg of *DPs* samples extracted by different methods, add 10 mL of deionized water, and dilute them in turn on the basis of complete dissolution, so that the final concentration of polysaccharides is 1, 2, 4, 8, and 10 mg/mL, and stored at 4 °C for later use.

#### 2.5.1 Determination of DPPH• scavenging ability

The DPPH• scavenging ability of *DPs* was determined according to the method studied by Mendes et al., but slightly modified. First, DPPH• solution (0.05 mmol/L) was prepared with ethanol (95%), and it was prepared and used now. Mix 1 mL of the polysaccharides solution well with 2 mL of DPPH (dissolved in 95% ethanol) and 1 mL of deionized water. After the mixture was reacted in the dark for 30 min, the absorbance value (*As*) was measured at 517 nm with UV–Vis spectrophotometer (UV-9100, Rayleigh Analytical Instruments, Beijing, China). 95% ethanol solution was used to replace the sample solution as the control (*Ac*), and 95% ethanol solution was used to replace the DPPH• solution (*Ab*) for the blank control. Calculate the DPPH free radical scavenging capacity of polysaccharides according to the following formula:

(2) $$\mathrm{Clearancerate}\left(\%\right)=1-\frac{A_{s}-A_{b}}{A_{c}}\times 100\%$$

#### 2.5.2 Determination of superoxide anion free radical scavenging ability

The superoxide anion free radical scavenging ability of *DPs* was determined according to the method studied by Zeng et al., but slightly modified. First, NADH, PMS and NTB were added to the solutions prepared by 16 mmol/L Tris-HCL buffer (pH 8.0) at 45 μmol/L, 108 μmol/L and 557 μmol/L, respectively. And take 1 mL of the above three solutions and 0.1 mL polysaccharides solution (2 mg/mL) and mix them well. After reacting the mixture in the dark for 5 min at 25 °C, the absorbance value (*As*) was measured at 560 nm with a UV–Vis spectrophotometer (UV-9100, Rayleigh Analytical Instruments, Beijing, China). Control group (*Ac*). The superoxide anion radical scavenging capacity of polysaccharides was calculated according to the following formula:

(3) $$Superoxideanionradicalscavengingrate\left(\%\right)=\frac{A_{C}-A_{S}}{A_{C}}\times 100\%$$

#### 2.5.3 Determination of hydroxyl radical (OH•) scavenging ability

The hydroxyl radical scavenging ability of *DPs* was determined according to the research method of Zhao et al., but slightly modified. 2.67 mmol of deoxyribose and 0.13 mmol of EDTA were dissolved in 0.2 mol/L PBS buffer (pH 7.4). And 0.6 mL PBS solution and 0.1 mL 2 mg/mL polysaccharides solution, 0.2 mL 0.4 mmol/L ferrous ammonium sulfate solution, 0.05 mL 2 mmol/L ascorbic acid solution and 0.05 mL 20 mmol/L H_2_O_2_ solution were thoroughly mixed. After incubating at 37 °C for 15 min, 1 mL of 1% thiobarbituric acid (w/v) and 2% trichloroacetic acid solution (w/v) were added to each. After the mixed solution was boiled for 10 min, it was rapidly cooled to room temperature, and the absorbance value was measured at 520 nm with an ultraviolet–visible spectrophotometer (UV-9100, Rayleigh Analytical Instruments Company, Beijing, China), with no sample added as a control (*Ac*). The hydroLI radical scavenging capacity of the polysaccharides was determined using the following formula:

(4) $$Hydroxylradicalscavengingrate\left(\%\right)=\frac{A_{C}-A_{S}}{A_{C}}\times 100\%$$

### 2.6 Preliminary characterization of different *Dictyophora indusiata* polysaccharides

#### 2.6.1 IR analysis of *Dictyophora indusiata* polysaccharides

Take 10 mg of the purified *DPs* samples extracted by different methods and add them to 50 times the mass of dry potassium bromide, after being fully ground, they were pressed into transparent sheets and placed in a Fourier transform infrared spectrometer (Bruker, Germany) for spectral measurement (Frequency range: 4000–500 cm^−1^) [15].

#### 2.6.2 NMR transverse relaxation

Measurement of transverse relaxation *T_2_* using the MesoQMR23-060H-I NMR analyze (Suzhou Niumag Analytical Instrument Co., Suzhou, China). The instrument is equipped with a 0.5-T permanent magnet, corresponding to a proton resonance frequency of 23 MHz at 32 °C. Dry samples were placed individually in cylindrical trays and place a 60 mm diameter radio frequency coil to collect the decay signal from the Carr-Purcell-Meiboom-Gill impulse train. The 90° and 180° pulse were 21.0 and 41.0 μs, respectively, and the *τ*-value (the time between the 90° and 180° pulses) is 100 μs. 500 echoes of data were collected as eight scan repetitions. The MultiExp Inv Analytical analysis software was used to analyze decay data.

#### 2.6.3 Molecular weight distribution of polysaccharides

The size exclusion chromatography combined with multi-angle laser scattering (HPSEC-MALLS) analysis determined the average molecular weight (*M*_w_), polydispersion index (PDI) and radius of rotation (〈*S*^2^〉_z_^1/2^) of polysaccharides in 0.1 mol NaCl solution. HPSEC-MALLS were measured by a multi-angle laser scatterometer (DAWN HELEOS, Wyatt Technology Co., Santa Barbara, CA, USA), CA, USA) on Agilent 1100 Series High Performance Liquid Chromatography system (Agilent Technologies, Palo Alto, CA, USA).The columns used were Ohpak SB-G (guard column, Shodex, Tokyo, Japan), Ohpak SB-806, SB-805 and SB-804 HQ columns (Shodex, Tokyo, Japan) selected in series at 35 °C. At the same time, refractive index detector was also connected (RID, Optilab rEX refractometer, DAWN EOS, Wyatt Technology Co., Santa Barbara, CA, USA). The specific refractive index increment (*dn/dc*) of the polysaccharide in 0.1 mol NaCl solution measured at 35 °C using a differential refractive index detector at a wavelength of 658 nm was 0.135 mL/g. The test mobile phase used 0.1 mol NaCl solution with a flow rate of 0.5 mL/min. All polysaccharide solutions (approximately 1 mg/mL) were filtered through a 0.22 μm filter, and the injection volume was 1000 μL. Astra software was used for data acquisition and analysis.

#### 2.6.4 Monosaccharide composition

Approximately 5 mg of *DPs* were hydrolyzed in a sealed tube with trifluoroacetic acid (2 M) at 121 °C for 2 h and dried with nitrogen. Then add methanol to wash and dry, repeat the step 2–3 times. The residue was reconstituted with deionized water, filtered through a 0.22 μm microporous membrane, and the filtrate was collected for testing. Polysaccharide extracts were analyzed by high-performance anion-exchange chromatography (HPAEC) with a CarboPac PA-20 anion-exchange column (3 × 150 mm; Dionex Co., California, USA) and a pulsed amperometric detector (PAD; Dionex ICS 5000 system). Flow rate, 0.5 mL/min; Injection volume, 5 μL; Solvent system A: (ddH_2_O), Solvent system B: (0.1 M NaOH), Solvent system C: (0.1 M NaOH, 0.2 M NaAc); Gradient program, the volume ratio of solutions A, B, C was 95:5:0 at 0 min, 85:5:10 at 26 min, 85:5:10 at 42 min, and 42.1 min at 60:0:40, 60:40:0 at 52 min, 95:5:0 at 52.1 min, 95:5:0 at 60 min. Data were processed using chromeleon 7.2 CDS (Dionex, Thermo Fisher Scientific, USA).

### 2.7 Statistical analysis

Data processing system (DPS) was used to analyze the single-factor data and Origin9.0 was used to make a graph. The data were analyzed by Design-Expert 8.0.6 software. The antioxidative activity of *DPs* was determined by parallel experiments and repeated three times, and the results were expressed as “mean ± standard deviation” (SD), using SPSS (Version 22, IBM Corporation, USA) for significant analysis of variance (ANOVA), when *p* < 0.05, the difference was considered significant and plotted with OriginPro 9.0.

## 3 Results and discussion

### 3.1 The yield of *Dictyophora indusiata* polysaccharides

Fig. S1(A) shows the extraction yields of DPS obtained by the four extraction methods, and the order is: UMAE > UAE > MAE > HWE. UMAE had the highest polysaccharides yield, while HWE is the lowest yield. UMAE has the highest extraction efficiency, which might be attributed to the effect of microwave radiation in part of it, which effectively destroys tissue and cell walls, resulting in the release of compounds into the solvent, allowing a larger contact area between the solid and liquid phases, and thus easier become a solvent for valuable components [5]; The cavitation effect of ultrasound could effectively disrupt the cell wall and enhance the exposure of the target compound to the solvent without changing the structure and molecular properties of the polysaccharide [25], [38].

### 3.2 Single factor test results of combined ultrasonic-microwave extraction

#### 3.2.1 Effect of extraction time on yield of *Dictyophora indusiata* polysaccharides

The effect of extraction time on DPs yield is shown in Fig. S1(B). The results showed that the DPs yield increased rapidly within 2–5 min, and the yield reached a peak at 6 min, followed by a slow decline. It might be because the effect of time is mainly reflected in the combined effect of microwave and ultrasonic waves. With the extension of time, under the influence of ultrasonic cavitation and microwave thermal effect, the material cells are rapidly ruptured, and the contents such as polysaccharides are released, so the yield The rapid increase and the slow decrease might be caused by the degradation of the polysaccharides due to the destruction of polysaccharides by microwave action. In addition, the thermal effect of microwaves continued to prolong with the extraction time, which might adversely affect the antioxidant activity of polysaccharides. Therefore, 6 min was chosen as the optimal extraction time.

#### 3.2.2 Effects of ultrasonic power on the yield of *Dictyophora indusiata* polysaccharides

Fig. S1(C) shows that the polysaccharides yield increased significantly when the ultrasonic power was increased from 150 to 540 W, and when the ultrasonic power continued to increase, the polysaccharides yield stabilized without a significant decrease. The above changes might be due to the enhancement of cell wall fragmentation with the increase of ultrasonic power, and the stability might be related to the effect of different extraction methods on the physical stability of polysaccharides. Considering the energy consumption issue comprehensively, it is advisable to use the ultrasonic power of 540 W as the best condition.

#### 3.2.3 The effect of microwave power on the yield of *Dictyophora indusiata* polysaccharides

The results are shown in Fig. S1(D), when the microwave power was increased from 40 to 130 W, the yield of *DPs* increased significantly, while when the microwave power increased from 130 to 160 W, the yield was almost stable and at 160 W, the yield of *DPs* reached the maximum value, and then continue to increase the power, but the yield decreases. The above changes are mainly attributed to the high-energy effect and strong penetrating ability of microwave radiation, which could quickly reach the interior of the material cells for heating, causing the cells to expand and rupture, and the content to flow out faster. The generated electromagnetic field is accelerated, and the strong microwave action would destroy the structure of the DPs, resulting in a decrease in the yield. In the UMAE system, 130 W is the optimum condition for microwave power.

#### 3.2.4 Effect of solid–liquid ratio on yield of *Dictyophora indusiata* polysaccharides.

Fig. S1(E) shows that the yield of DPs increases significantly as the solid-to-liquid ratio increases from 10 to 40 mL/g, while the yield decreases from 40 to 70 mL/g. These changes might be attributed to two aspects: on the one hand, too low material-to-liquid ratio is not conducive to material dispersion and moisture penetration, resulting in lower yields; on the other hand, too high moisture content could delay the thermal effect of microwaves and weaken ultrasonic wave’s effect. Taking into account comprehensively, 40 mL/g was adopted as the optimal material-liquid ratio condition.

### 3.3 Response surface test results of ultrasonic-microwave combined extraction

#### 3.3.1 Model fitting and data analysis

On the basis of single sfactor experimental analysis, the effect of the interaction of four independent variables of solid–liquid ratio, extraction time, ultrasonic power and microwave power on the yield of DPs was studied through 30 random sequence experiments. Table S1 shows the design level and corresponding response surface design results. The data generated by the BBD experiment was analyzed by multiple regression analysis using Design-Expert 8.0.6, and the quadratic polynomial equation between the polysaccharides yield and the extracted variable was obtained as follows:

(5) $$Y=12.61+0.23X_{1}+0.16X_{2}+0.071X_{3}-0.023X_{4}-0.11X_{1}X_{2}-0.094X_{1}X_{3}-0.036X_{1}X_{4}+0.14X_{2}X_{3}-0.20X_{2}X_{4}-0.091X_{3}X_{4}-0.25X_{1}^{2}-0.23X_{2}^{2}-0.15X_{3}^{2}-0.18X_{4}^{2}$$

In formula (5) shown above, *Y* is the yield of *DPs* (%), and *X_1_*, *X_2_*, *X_3_*, and *X_4_* are the extraction time, ultrasonic power, microwave power, and solid–liquid ratio, respectively. The corresponding *F*-value and *P*-value were used to test the response surface quadratic model analysis of variance, and the results are shown in Table S2. The results of significant variance analysis showed that the *F* value of the quadratic regression model was 56.04 (*p* < 0.0001), indicating that the regression model was statistically significant. The lack-of-fit value relative to the pure error is not significant within the 95% confidence interval, indicating the actual availability of the model. The coefficient of determination (*R^2^*) is an index to test the quality of the model. A high coefficient of determination could ensure that the quadratic model makes corresponding adjustments to the experimental data. The model *R^2^* = 0.9812, *Adj-R^2^* = 0.9637, indicating that the model has a good fit spend.

The order of the effects of the four independent variables is *X_1_* > *X_2_* > *X_3_* > *X_4_*, which indicated that the extraction time (*X_1_*) has the greatest effect on the DPs yield (*p* < 0.0001), while the solid–liquid ratio has no significant effect on the PID (*p* > 0.05). *X_1_X_2_*, *X_1_X_3_*, *X_2_X_3_*, *X_2_X_4_*, *X_3_X_4_*, etc.,the interaction conditions also showed a significant effect on the yield of DPs (*p* < 0.05).

#### 3.3.2 Response surface and contour plot analysis of DPs

According to the previous drawing, the response surface analysis diagram of the interaction of each factor in the model is drawn, and the response surface diagram and corresponding contour diagram of each interaction are obtained as shown in Fig. S2. After any two factors of ultrasonic power, microwave power, solid–liquid ratio and extraction time were fixed at the level of one of the factors, the change trend of the polysaccharides yield of *Dictyophora indusiata* with the increase of the other factor was that it first increased and then decreased, probably because the strong microwave-ultrasonic condition damages the structure of polysaccharides, resulting in the degradation of polysaccharides and the decrease of the yield.

#### 3.3.3 Response surface and contour plot analysis of DPs

In order to obtain the highest response value of *DPs* yield, the model conditions were optimized by Design-Expert 8.0.6, and the optimal conditions for the extraction of *DPs* were obtained as follows: extraction time 6 min, microwave power 150 W, ultrasonic power 550 W, feed liquid Compared with 45 mL/g, the yield of *DPs* was 12.66%. Through the verification test, under the same conditions, the polysaccharides yield of *Dictyophora indusiata* measured in parallel experiments was 12.36%, which was close to the predicted value, indicating that the model was feasible and accurate for predicting the yield of *DPs*.

### 3.4 Analysis of surface morphology of *Dictyophora indusiata*

**Fig. 1:**
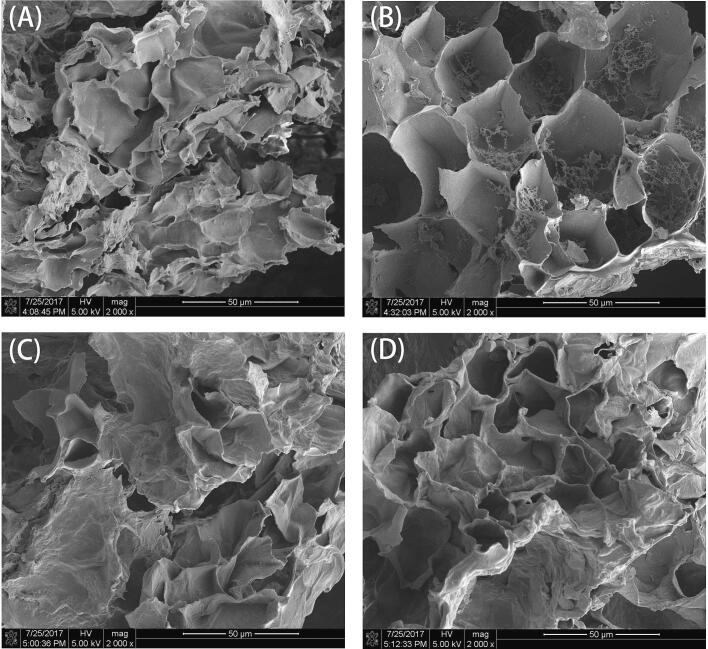
Scanning electron microscope of DPs.

The morphological changes of UAE, MAE, HWE and UAME were characterized by scanning electron microscopy, and the results are shown in the Fig. 1 (2000 times). After four different extraction methods, the *Dictyophora indusiata* had different degrees of dispersion and wrinkles on the surface. It could be observed from the Fig. S2 that the surface from polysaccharides extracted by UAE presents a complete honeycomb shape, the surface is relatively smooth and aggregated into clusters. After UAME treatment, it was found that the surface has a partial honeycomb shape, and the degree of surface collapse and fold is more obvious than other extraction methods, thereby accelerating the dissolution of cells. After the MAE treatment, the surface of *Dictyophora indusiata* has no obvious honeycomb shape, and the tissue shape begins to break, with many wrinkles and rough surface. However, after HWE extraction, most of the surfaces showed irregular folds and rough surfaces, without obvious honeycomb shape and with holes in larger blocks. Compared with the other three extraction methods, the damage to the tissue structure was the most serious. According to the antioxidant activity, the strong oxidative effect of the polysaccharides from *Dictyophora indusiata* might be attributed to the honeycomb shape of UAME, UAE, and MAE, which might make the active sites of polysaccharides fully exposed and contributes to their role in antioxidant activity [20], [21].

### 3.5 Effects of different extraction methods on antioxidant activity of *Dictyophora indusiata* polysaccharides in vitro

#### 3.5.1 Comparison of DPPH• scavenging ability of polysaccharides extracted by different methods

**Fig. 2:**
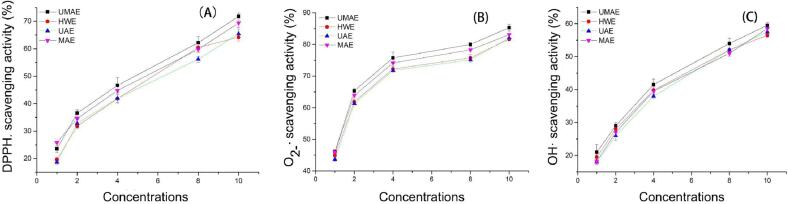
Effect of extraction methods on scavenging activity (A) DPPH•, (B) O_2_^−^, (C) OH•.

DPPH• method is a common method for evaluating antioxidant activity. Antioxidant substances could directly act on DPPH• free radicals to lighten the color. The effect of different extraction methods on DPPH• scavenging activity is shown in the Fig. 2(A). All four polysaccharides showed a dose–effect relationship between mass concentration and free radical scavenging, that is, within a certain mass concentration range, with the increase of polysaccharides mass concentration, its scavenging effect on DPPH free radicals also increased The DPPH• clearance rates of UMAE, HWE, UAE and MAE increased with the increase of polysaccharides concentration in the range of 1–10 mg/mL polysaccharides concentration, showing an obvious dose-dependent manner. When the concentration is 1 mg/mL, the scavenging rate of DPPH• radicals by four methods is 18%-24%, and the scavenging rate of MAE method is the highest. When the concentration continued to increase, UMAE was the highest. When the concentration increased to 10 mg/mL, the scavenging rate of DPPH• radicals by four methods was 72–77%, and the scavenging rate of DPPH• radicals by UMAE extraction method was higher than that of the other three methods, which might be due to the ultrasonic-microwave extraction method degrades the smaller molecular weight polysaccharides fractions of *Dictyophora indusiata* with solubility increasing [38], exposes more active groups, and easily captures DPPH•. In addition, when the polysaccharides concentration was 8–10 mg/mL, the hot water extraction method did not significantly enhance the DPPH free radical scavenging rate, which might be due to the fact that the hot water extraction method did not increase the dissolution of polysaccharides.

#### 3.5.2 Comparison of superoxide radical scavenging ability of polysaccharides extracted by different methods

Superoxide anion radicals are weak oxidants, but binding to hydroxyl molecules could damage DNA and other biomolecules. The Fig. 2(B) shows that effects of different extraction methods on superoxide anion radical scavenging activity. In the polysaccharide concentration range of 1–10 mg/mL, the clearance rates of the four extraction methods increased with the increase of polysaccharide concentration. When the concentration of DPs is 1 mg/mL, the superoxide anion scavenging rate of the four extraction methods is about 44%. When the polysaccharide concentration exceeds 2 mg/mL, with the increase of the concentration of polysaccharides but decreased scavenging ability. The scavenging rate of DPs extracted by four extraction methods UMAE, HWE, UAE, and MAE to scavenge superoxide anion free radicals increases slowly. When the concentration is 10 mg/mL, the maximum scavenging rate reaches 83.23%, 81.45%, and 81.75% respectively and 82.57%, among which UMAE has the highest removal ability.

#### 3.5.3 Comparison of OH• scavenging ability of polysaccharides extracted by different methods

OH• is a highly active free radical produced in the body in a short time, which is very harmful to the organism. It is considered to be an effective reaction substance that could promote the oxidation of functional biological macromolecules in living cells [17]. The effect of the same extraction method on OH• scavenging activity is shown in the Fig. 2(C). The scavenging effects of UMAE, HWE, UAE and MAE on OH• were positively correlated with the polysaccharides concentration in the range of 1–10 mg/mL polysaccharides concentration. When the polysaccharides concentration was 1 mg/mL, the scavenging rates of OH• by the four methods were all lower than 20%. When the concentration increased to 10 mg/mL, the scavenging rates of OH• by the four methods were about 60%. OH• has the strongest scavenging rate. The mechanism by which polysaccharides scavenge hydroxyl radicals is related to the interaction between hydroxyl hydrogen and free radicals and the termination of the free radical chain reaction. However, the mechanism has not been efficiently described [10].

#### 3.5.4 Comparison of antioxidant activity of polysaccharides extracted by different methods

The antioxidant activity of *DPs* was measured by the investigation of DPPH• scavenging ability, superoxide anion free radical scavenging ability and hydroxyl radical (OH•) scavenging ability. It could be seen from the data that different extraction methods also have differences in the antioxidant activity of *DPs*, and the different antioxidant activities might be related to the differences in the chemical composition and molecular properties of polysaccharides caused by different extraction methods [6]. Among them, DPPH• scavenging ability, superoxide anion free radical scavenging ability, hydroxyl radical (OH•) scavenging ability is the highest scavenging ability of UMAE whether at high or low polysaccharides concentration (except DPPH• scavenging ability at 1 mg /mL), so the polysaccharides extracted from UAME had the highest antioxidant activity. There were might be due to two reasons: one is due to the exposure of more groups; the other is due to reports that the antioxidant activity increases with the decrease of the molecular weight of polysaccharides, while the average molecular weights of UAM and MAE extracted polysaccharides are both low [22]. At the same time, UMAE is a complementary technology that combines the advantages of both, which not only shortens the extraction time, but also makes its polysaccharides have high antioxidant properties.

### 3.6 Structural characterization of different *Dictyophora indusiata* polysaccharides

#### 3.6.1 IR analysis of *Dictyophora indusiata* polysaccharides

**Fig. 3:**
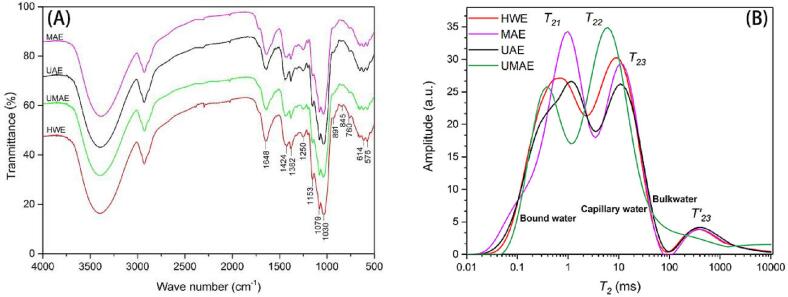
FT-IR spectra (A) and low field nuclear magnetic resonance (B) of DPs by different extraction methods.

FT-IR spectroscopy is an effective technique for identifying characteristic organic groups in polysaccharides [41]. Four kinds of polysaccharides obtained from UAE, MAE, UAME and HWE were analyzed by infrared spectrum scanning, and the results are shown in the Fig. 3(A). The infrared spectra of polysaccharides obtained by different extraction methods are basically similar, and only differ in the intensity of the bands. Li et al. found that typical polysaccharides characteristic absorption peaks at 3420, 2920, 1620, 1400 and 1100 cm [16]. The broad and strong absorption peak around 3400 cm^−1^ should be the O-H stretching vibration of sugars [18], which indicated that there are intermolecular and intramolecular hydrogen bonds in all four polysaccharides. The absorption peak in the range of 2800 ∼ 3000 cm^−1^ is generally caused by the C–H stretching vibration of –CH_2_– of polysaccharides [30]. The weaker absorption peak around 2300 cm^−1^ is generally caused by the presence of CO_2_ in the test environment. The absorption peaks at 1648 and 1424 cm^−1^ lead to the existence of carboxyl and carbonyl groups, respectively [36], and the absorption peak near 1648 represents the C=O asymmetric stretching vibration absorption band [32]. The peak near 1250 cm^−1^ is due to the S=O symmetric stretching vibration [24]. The absorption peak around 1030 cm should be attributed to the characteristic peak of pyranoside. In addition, each polysaccharides has a specific band in the range of 1000–1200 cm^−1^, and the absorption in this region is attributed to the stretching vibration of C–O–C and C–OH side groups [3]. In addition, furanosides are characterized by absorption at approximately 1079 cm^−1^ [23]. The polysaccharides obtained by four extraction methods exhibit characteristic absorption at 845 cm^−1^, and the polysaccharides treated with HWE has the strongest absorption peak, indicating that its constituent monosaccharides include pyranose and obvious α-glycosidic bonds. 891 cm^−1^ is *β*-glycosidic bond, which is correspond to the *β*-glucan structures of polysaccharides [9]. But after the HWE treatment, the polysaccharides has the strongest absorption peak. Its structures was affected after ultrasonic and microwave treatment [14]. The absorption at 900 ∼ 500 cm^−1^ might be related to the absorption skeleton mode of the pyranose ring [35], so 576 cm^−1^ should be the characteristic absorption peak of the pyranose ring. These results show that the polysaccharides from different extraction methods have typical polysaccharides absorption peaks, and different extraction methods have no significant effect on the types of glycosidic bonds and sugar rings of polysaccharides [8].

#### 3.6.2 NMR transverse relaxation measurements

LF-NMR cuold measure the distribution of hydrogen protons with different relaxation times, and could be used to predict the migration of various water in substances based on the difference in proton relaxation. These provide information on the moisture mobility, distribution and internal structural changes of the extracted polysaccharides powder. The proton spin–lattice (*T_1_*) and relaxation time and spin–spin (*T_2_*) is closely related to the water content of the substance, the physical properties of various types of water, and the binding state of the interaction between water and macromolecules [39]. The relaxation decay curves of *DPs* obtained by different extraction methods are shown in the Fig. 3(B) and Table 1.

**Table 1:** The quantitative data of *T*_2_ values and molar mass ratio of monosaccharide in different extracted methods.

Extraction method	HWE	MAE	UAE	UMAE
*T_21_*(ms)	0.756	1.000	1.150	0.376
*A_21_*(%)	48.990	57.409	55.972	33.963
*T_22_*(ms)	9.326	10.723	10.723	6.136
*A_22_*(%)	45.038	36.915	37.453	64.128
*T_23_*(ms)	351.119	403.702	403.702	10000.000
*A_23_*(%)	5.972	5.676	6.576	1.909

Fuc	1.000	1.000	1.000	1.000
Ara	0.119	0.112	0.130	0.080
Rha	–	–	–	–
Gal	4.889	4.998	4.952	5.011
Glc	73.224	75.401	82.802	56.231
Xyl	0.126	0.092	0.139	0.073
Man	6.664	5.797	13.338	4.621
Fru	–	–	–	–
Gal-UA	–	–	–	–
Glc-UA	0.270	0.213	0.247	0.142

*T_21_* and *T_22_* indicate the decay time of the first, second and third peak in each sample, respectively. *A_21_*, *A_22_* and *A_23_* means the percentage of peak area for each sample.

*Fuc, Ara, Rha, Gal, Glc, Xyl, Man, Fru, Gal-UA, Glc-UA denote Fucose, Arabinose, Rhamnose, Galactose, Glucose, Xylose, Mannose, Fructose, Galacturonic Acid, Glucuronic Acid. “–” means no detection.

Decay highly depends on water content. A distribution of relaxation times was obtained in the multi-exponential fitting of the decay data. The water groups of three different dried polysaccharides powders could be identified from the relaxation spectra at *T_2_* in the Fig. 3(B). Polysaccharides extracted by all four methods had signals at <1.5 ms (*T_21_*), which demonstrates that the strongly bound water is tightly bound to the polar groups on the surface of the polysaccharides. The combined water content of MAE was the highest and UMAE was the least. The second proton population with signals in the range of 2–10 ms (*T_22_*) was the lightly bound water associated with highly organized structures or trapped in the tissues [26]. UMAE and HWE retain some lightly bound water with polysaccharides. Compared to the other two forms of water, free water absorbs magnetic energy more easily to vibrate, and takes longer to return to its ground state after the magnetic field has disappeared. The third (*T_23_*) and fourth (*T'_23_*) peaks of each polysaccharides have longer relaxation times, which were the relaxation peaks of free water [26]. A signal of 10–100 ms (*T_23_*) is considered to the immobilized water within polysaccharides lattice [1]. It was found in both UAE and MAE polysaccharides, indicating the presence of lightly bound fixed water on the surface of the polysaccharides molecules. Furthermore, UMAE has no proton signal in the range of 100–1500 ms (*T_23_*), which indicates that there is no free water on the surface of polysaccharides after drying.

In general, the relaxation time of the fixed water and the bound water of UMAE polysaccharides decreased, which might increase the hydrophilic ability of polysaccharides and retain a higher content of fixed water that was lightly bound to polysaccharides. The relaxation times of fixed and bound water for UAE and MAE was higher, and the hydrophilic ability of polysaccharides was decreased. Compared with hot water extraction, UAE converts the fixed water of MAE to bound water and free water, and MAE promotes the conversion of fixed water to bound water. The water retention might be related to the composition, molecular weight and molecular conformation of polysaccharides. Shorter molecular chains have better hydrophilicity, and higher molecular weights (complete polysaccharides molecular structure) have the stronger hydrophobicity. It has been shown that ultrasound might cause structural degradation of polysaccharides, which in turn leads to the formation of more free water, and might contribute to the process of rehydration of dried polysaccharides (increased solubility). Interestingly, the application of combined microwave and ultrasonic extraction treatments might help to reduce the damage to polysaccharides structure caused by ultrasound. Polysaccharides treated by UMAE showed stronger antioxidant activity, which might be due to the presence of lightly bound water in the immobilized state promoting functional group activity, thus making it easier to reach the free water molecules during solubilisation and driving the formation of new reduction reactions.

#### 3.6.3 Absolute molecular weight and molecular conformation

**Fig. 4:**
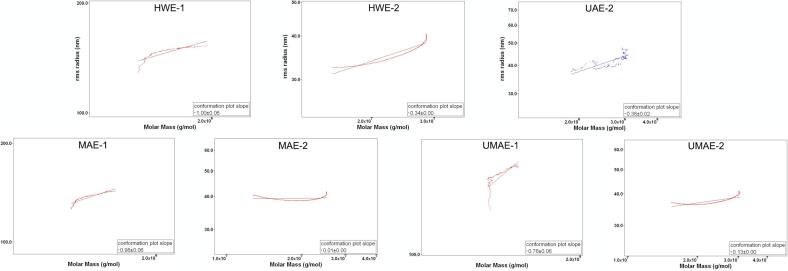
Molecular conformation of DPs.

The *Mw/Mn* values of each component of polysaccharides extracted by the four methods were close to 1, indicating that each component was homogeneous polysaccharides. Among them, the hot water extracted fraction 1 (HWE-1) had the highest weight average molecular weight and mean square rotation radius of all extracted polysaccharides at Table 1. By *r*_g_ plotting against *Mw*, HWE-1 had a slope of 1 and a rigid rod conformation, indicating that it probably exist in water as a linear structure consisting of a triple helix conformation. Component 2 (HWE-2) has the lowest mean square rotation radius and a slope of 0.34, indicating a spherical conformation (Fig. 4).

There is only one component in UAE extraction. The slope of UAE-2 is 0.38, and it has a spherical conformation. At the same time, it presents a partial U-shape and a partial branching structure in the conformation. MAE-1 has a slope of 0.98 and is a rigid rod-like conformation. The slope of MAE-2 is 0.01 and is a solid sphere conformation. UMAE-1, with a slope of 0.76, although still in a rod-like conformation, it has been transformed like an irregular linear cluster, indicating that the structure of the polysaccharides might be extended, thus exposing the hydroxyl group, which helps to fix the formation of water. The slope of UMAE-2 is 0.13, with a conformation shift to a solid sphere compared to the hot water extraction. Interestingly, although both MAE-2 and UMAE-2 shifted from a hollow sphere to a solid sphere state (molecular weight increased, but UMAE-2 did not form solid sphere structure). However, the water morphology tended to form bound and fixed water respectively, suggesting that the formation of bound water might be caused by the formation of short polysaccharides molecular chains formed by the degradation of polymer chains and then transformed into fiber networks. It is also related to the formation of crystal bound water (due to the low molecular weight, the detector did not detect the formation mechanism of MAE-2 and UAE-2 bound water is similar).

Only use MAE and UAE resulted in degradation of polysaccharides molecular chains and the formation of short molecular chains, which might contribute to the formation of helices and assembly into crystal structure, which is not conducive to the antioxidant activity of polysaccharides. However, the retention of the high molecular weight fraction (MAE-1) of *DPs* by MAE extraction might be a key determinant of the difference in antioxidant effect with UAE (degradation of high molecular weight fraction UAE-1).

The free radical scavenging ability of polysaccharides was significantly affected by the extraction methods, although they had the same basic structure and advanced structure. There were different water attachment modes in the polysaccharides molecules. The polysaccharides extracted by UMAE contributed to the moderate extension of the structure of the high molecular weight components of *DPs*, and tended to form the polysaccharides with long medium chain structure rather than short chain structure, which enhanced the retention of fixed water. There were more retained lightly bound water retained on the molecular surface, but the immobilized water and free water were lower. The difference in free radical scavenging ability might be due to the presence of fixed water, which might promote the arrival of functional groups to free water molecules and contribute to the enhancement of antioxidant activity.

#### 3.6.4 Analysis of monosaccharide composition

The moles of monosaccharides detected in HWE, UAE, MAE and UMAE are shown in Table 2, and the main monosaccharide in DPs is Glc. The types of monosaccharides in DPs were not affected by different extraction methods, but the molar ratio was significantly different. Glc is the main monosaccharide, along with Man and Gal and, in turn, small amounts of Fuc, Ara, Xyl and GLC-UA. *Dictyophora indusiata* has been found to consist of Glc (56.2%), Man (29.7%) and Gal (14.1%). The glycosidic bond connection type are *β*-1,6-Glc*p*, *α*-1,2,6-Glc*p*, *α*-1,3-Man*p*, *β*-1,6-Man*p* and *β*-1,6-Gal*p*, Among them, *α*-1,3-Mannan and *β*-1,3-Glucan are the main components of hot water extraction fractions obtained [19]. Consistent with the type of monosaccharide composition type of this study with higher Glc and lower ratio of Man and Gal, the differences might be related to the origin of the raw materials. The results showed that the DPs extracted mainly consisted of dextran.

**Table 2:** Molecular weight and molecular conformation.

Method	Name	Mass Fraction (%)	Molar mass moments (g/mol)		Polydispersity	Rms radius moments (nm)	
			*Mw*	*Mn*	*Mw/Mn*	*Rw*	*Rn*
HWE	Fraction 1(HWE-1)	43.1	1.841 × 106 (±0.705%)	1.839 × 106 (±0.678%)	1.001 (±0.292%)	146.2 (±0.6%)	146.1 (±0.6%)
	Fraction 2(HWE-2)	56.9	2.296 × 107 (±0.187%)	2.212 × 107 (±0.180%)	1.038 (±0.259%)	35.3 (±0.6%)	34.9 (±0.6%)
UAE	Fraction 2(UAE-2)	100	2.693 × 106 (±0.184%)	2.640 × 106 (±0.538%)	1.020 (±0.721%)	41.8 (±1.0%)	41.5 (±1.1%)
MAE	Fraction 1(MAE-1)	57.5	1.707 × 106 (±0.329%)	1.705 × 106 (±0.304%)	1.001 (±0.762%)	136.5 (±0.6%)	136.3 (±0.6%)
	Fraction 2(MAE-2)	44.2	2.048 × 107 (±0.220%)	1.957 × 107 (±0.209%)	1.047 (±0.304%)	39.4 (±0.5%)	39.4 (±0.5%)
UMAE	Fraction 1(UMAE-1)	50.3	1.536 × 106 (±0.493%)	1.534 × 106 (±0.471%)	1.001 (±0.995%)	125.1 (±0.6%)	125.0 (±0.6%)
	Fraction 2(UMAE-2)	49.7	2.411 × 107 (±0.212%)	2.308 × 107 (±0.198%)	1.045 (±0.290%)	37.6 (±0.5%)	37.4 (±0.5%)

Different letters in the same row represent significant differences between samples (*p* < 0.05).

*Mw* and *Mn* refer to weight-, number-average molecular weight, respectively. *Rw* and *Rn* refer to weight-, number-average square mean radius of gyration, respectively.

In addition, it has been found that one component contained Man, Glc, Gal and Xyl with a main chain was composed of → 1)-Glc-(6 → 1)-Man-(3,6 → 1)-Xyl-(5 → 1)-Gal-(3 → 1)-Gal-(6→: with the ratio of 4.9: 15.5: 7.8: 1.0: 5.7, and a relative molecular weight of 18.16 kDa, the other component is composed of Glc and Man, the main chain was composed of → 1)-Glc-(6 → 1)-Man-(3,6 → with the ratio of 5.6:1.0, the molecular weight is 2100 kDa [13]. These phenomena are similar to the properties of polysaccharides extracted by different methods. Combined with the analysis of Mass Fraction (%) and monosaccharide composition analysis in molecular weight analysis. It indicates that the first component extracted by each extraction method with relatively small absolute molecular weight might be Mannan (polysaccharides is rod-like configuration). The second component Glucan (polysaccharides have spherical configuration). In conclusion, the moderate structural extension of Mannan by UMAE and MAE extraction process (some release of the rod-like rigid helical conformation into a flexible conformation) might be the main factor that enhances the antioxidant activity of DPs.

## 4 Conclusion

In this study, the water-soluble polysaccharides of *Dictyophora indusiata* were obtained by four methods: ultrasonic-assisted extraction, microwave-assisted extraction, ultrasonic-microwave-assisted extraction and hot water-assisted extraction, and were optimized by BBD method. The optimal conditions for DPs extraction were established: extraction time 6 min, microwave power 150 W, ultrasonic power 550 W, solid–liquid ratio 45 mL/g, and the yield of *DPs* was 12.66%, which was close to the expected output value. In addition, the infrared spectra of the polysaccharides obtained by the four extraction methods were basically similar, and only differed in the intensity of the bands. Scanning electron microscope showed that ultrasonic-assisted treatment and ultrasonic-microwave-assisted treatment presented a relatively smooth honeycomb shape, which had a greater degree of damage to the cell wall of *DPs*. All four polysaccharides have certain antioxidant capacity, among which UAME has the best comprehensive antioxidant capacity. In addition, the mechanism of antioxidative activity of bamboo sunflower remains to be further studied. The molecular weight of DPs is significantly reduced after microwave or ultrasonic treatment, and the molecular weight of ultrasonically extracted DPs is reduced more and more short chains are formed. After drying, the intermolecular triple helix structure is easier to assemble and self-assemble into small pieces of rigid short fiber network supramolecular structure under relatively high concentration system environment, and more free water would be generated at the same time. Microwave molecular weight decreased (compared with ultrasonic extraction, macromolecular structure was better protected, long medium molecular chains were partially degraded, but many short molecular chains were generated), and intermolecular entanglement and fiber structure were also found. The combined microwave-ultrasonic treatment was beneficial to the transformation of DPs molecules from long chain to medium chain, and promoted the structural unfolding and exposure of more hydroxyl groups of mannans, generating in less short chain formation and more fixed water. It also avoids intermolecular helical and helps to lock the free water.

## CRediT authorship contribution statement

**Yanlin Zhang:** Investigation, Writing – original draft, Writing – review & editing. **Yi Lei:** Writing – original draft, Writing – review & editing. **Shirong Qi:** Methodology, Conceptualization. **Mingxuan Fan:** Visualization. **Shuyi Zheng:** Project administration, Resources, Software. **Qingbin Huang:** Funding acquisition. **Xu Lu:** Formal analysis, Supervision.

## Declaration of Competing Interest

The authors declare that they have no known competing financial interests or personal relationships that could have appeared to influence the work reported in this paper.
