# Supplementary material for: Ultrasonic-microwave-assisted extraction for enhancing antioxidant activity of Dictyophora indusiata polysaccharides: The difference mechanisms between single and combined assisted extraction
Source: Ultrason Sonochem. 2023 Mar 6;95:106356. doi: 10.1016/j.ultsonch.2023.106356 (PMC10014295; doi:10.1016/j.ultsonch.2023.106356)
Supplement: Supplementary data 1 [file mmc1.doc]

**
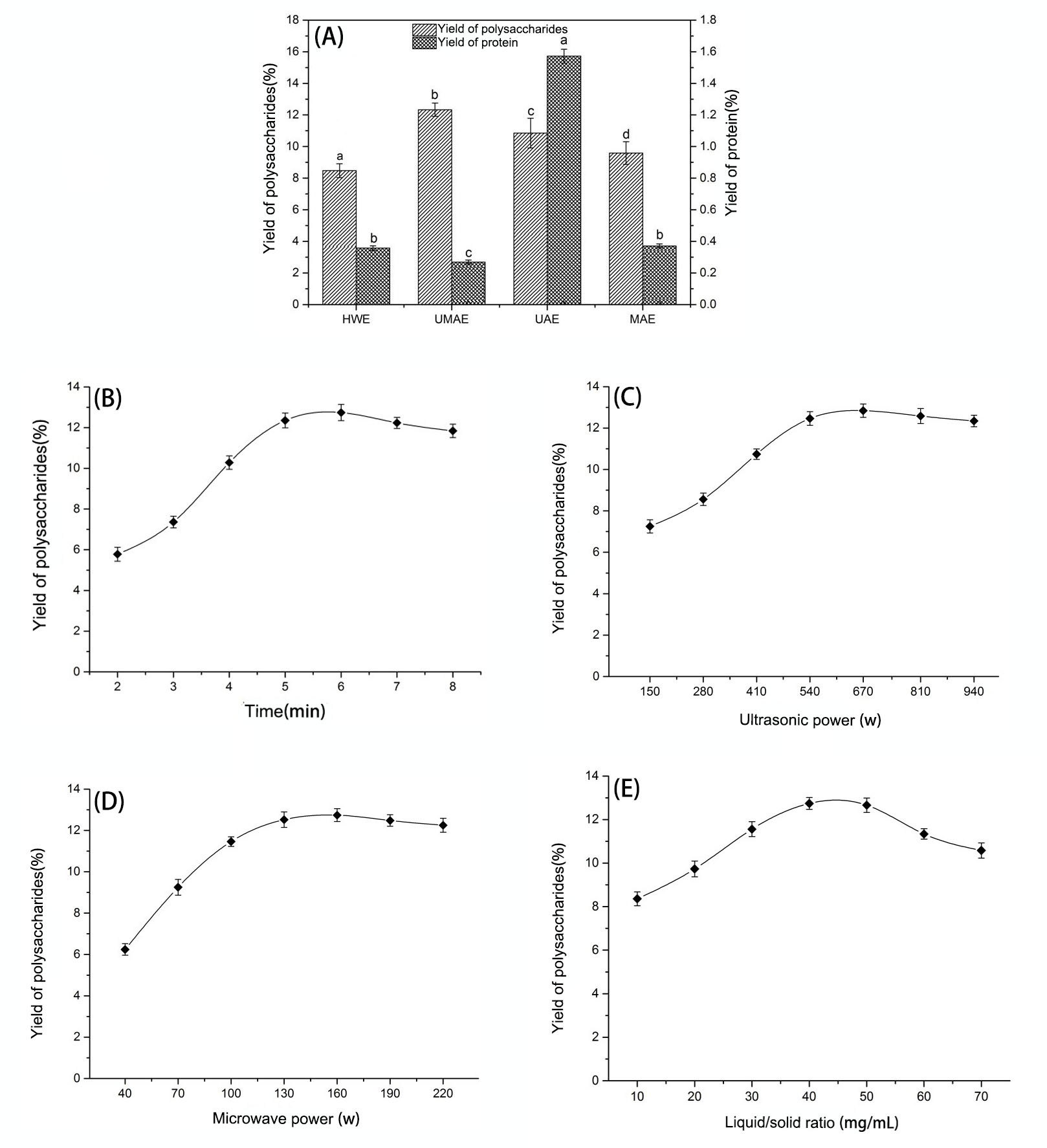
**

**Fig. S1.** Effect of extraction methods on the yield of DPs (A) Yield of polysaccharides and proteins, (B) Extraction time, (C) Ultrasonic power, (D) Microwave power, (E) Lipid/solid ratio.

**
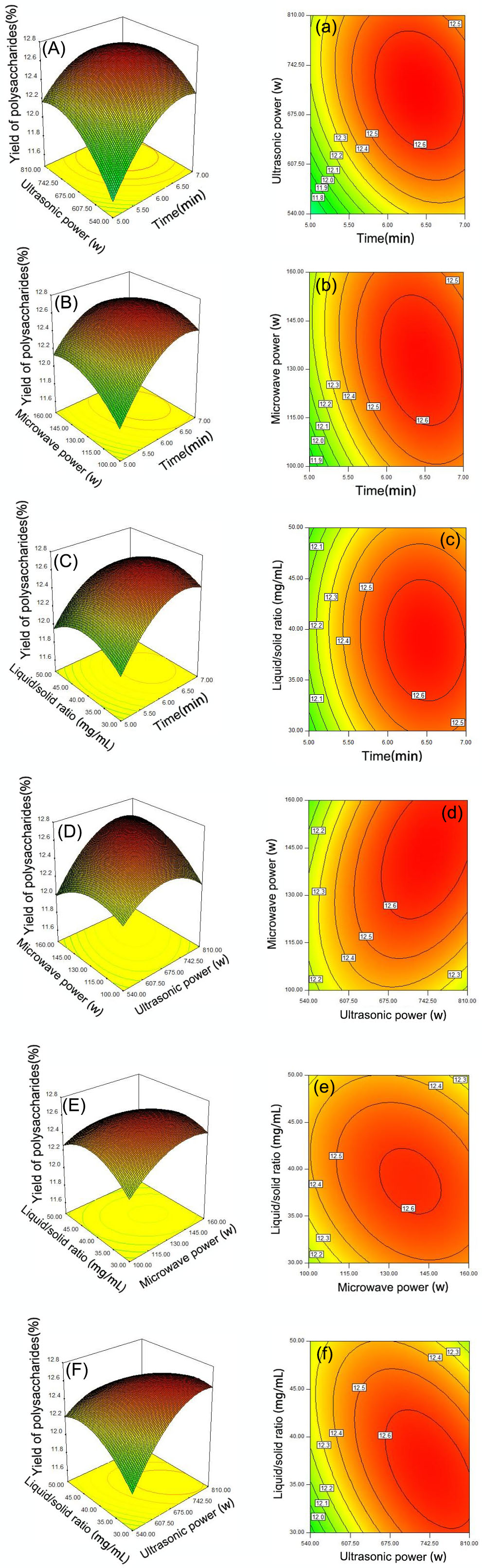

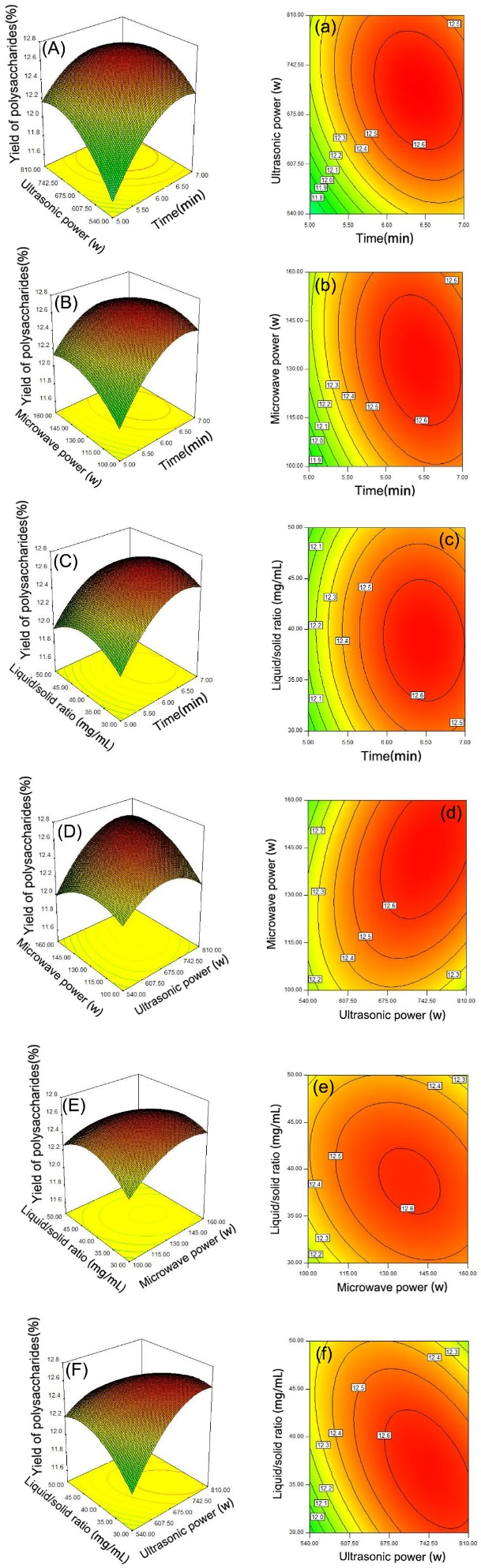
**

**Fig. S2.** Response surface plots and contour plots of factors mutual interactions on the yield of DPs.

**Table S1 CCD and result for extraction yield of DPs**

| Test number | Factor | | | | | Level | | | | Yield (%) |
| --- | --- | --- | --- | --- | --- | --- | --- | --- | --- | --- |
|  | *X_1_* | *X_2_* | *X_3_* | *X_4_* | *X_1_* | | *X_2_* | *X_3_* | *X_4_* |  |
| 1 | -1 | -1 | -1 | -1 | 5.0 | | 540 | 100 | 30 | 11.06 |
| 2 | 0 | 0 | 0 | 0 | 6.0 | | 675 | 130 | 40 | 12.57 |
| 3 | 0 | 0 | -2 | 0 | 6.0 | | 675 | 70 | 40 | 11.78 |
| 4 | +1 | -1 | +1 | -1 | 7.0 | | 540 | 160 | 30 | 11.75 |
| 5 | -1 | -1 | -1 | +1 | 5.0 | | 540 | 100 | 50 | 11.66 |
| 6 | +1 | -1 | +1 | +1 | 7.0 | | 540 | 160 | 50 | 11.92 |
| 7 | 0 | 0 | 0 | -2 | 6.0 | | 675 | 130 | 20 | 11.84 |
| 8 | 0 | +2 | 0 | 0 | 6.0 | | 945 | 130 | 40 | 12.1 |
| 9 | +1 | +1 | +1 | +1 | 7.0 | | 810 | 160 | 50 | 11.67 |
| 10 | 0 | 0 | 0 | +2 | 6.0 | | 675 | 130 | 60 | 11.92 |
| 11 | 0 | 0 | +2 | 0 | 6.0 | | 675 | 190 | 40 | 12.24 |
| 12 | +1 | -1 | -1 | +1 | 7.0 | | 540 | 100 | 50 | 12.32 |
| 13 | -1 | +1 | -1 | +1 | 5.0 | | 810 | 100 | 50 | 11.38 |
| 14 | +1 | +1 | -1 | -1 | 7.0 | | 810 | 100 | 30 | 12.15 |
| 15 | 0 | 0 | 0 | 0 | 6.0 | | 675 | 130 | 40 | 12.62 |
| 16 | 0 | 0 | 0 | 0 | 6.0 | | 675 | 130 | 40 | 12.52 |
| 17 | -2 | 0 | 0 | 0 | 4.0 | | 675 | 130 | 40 | 11.12 |
| 18 | +1 | -1 | -1 | -1 | 7.0 | | 540 | 100 | 30 | 11.94 |
| 19 | -1 | -1 | +1 | -1 | 5.0 | | 540 | 160 | 30 | 11.22 |
| 20 | +2 | 0 | 0 | 0 | 8.0 | | 675 | 130 | 40 | 12.15 |
| 21 | -1 | +1 | +1 | -1 | 5.0 | | 810 | 160 | 30 | 12.42 |
| 22 | +1 | +1 | -1 | +1 | 7.0 | | 810 | 100 | 50 | 11.81 |
| 23 | 0 | - 2 | 0 | 0 | 6.0 | | 405 | 130 | 40 | 11.32 |
| 24 | 0 | 0 | 0 | 0 | 6.0 | | 675 | 130 | 40 | 12.68 |
| 25 | -1 | +1 | +1 | +1 | 5.0 | | 810 | 160 | 50 | 11.89 |
| 26 | -1 | +1 | -1 | -1 | 5.0 | | 810 | 100 | 30 | 11.64 |
| 27 | -1 | -1 | +1 | +1 | 5.0 | | 540 | 160 | 50 | 11.35 |
| 28 | 0 | 0 | 0 | 0 | 6.0 | | 675 | 130 | 40 | 12.65 |
| 29 | +1 | +1 | +1 | -1 | 7.0 | | 810 | 160 | 30 | 12.52 |
| 30 | 0 | 0 | 0 | 0 | 6.0 | | 675 | 130 | 40 | 12.62 |

**Table S2** Analysis of variance for the response surface of yield extraction

| Item | Sum of squares | Degrees of freedom | Mean square | *F* Value | *P* Value(Salience) |
| --- | --- | --- | --- | --- | --- |
| Regression model | 6.83 | 14 | 0.49 | 56.04 | < 0.0001^**^ |
| *X_1_* | 1.27 | 1 | 1.27 | 145.94 | < 0.0001^**^ |
| *X_2_* | 0.61 | 1 | 0.61 | 69.89 | < 0.0001^**^ |
| *X_3_* | 0.12 | 1 | 0.12 | 13.84 | 0.0021^**^ |
| *X_4_* | 0.012 | 1 | 0.012 | 1.40 | 0.2557 |
| *X_1_X_2_* | 0.21 | 1 | 0.21 | 23.80 | 0.0002^**^ |
| *X_1_X_3_* | 0.14 | 1 | 0.14 | 16.16 | 0.0011^**^ |
| *X_1_X_4_* | 0.021 | 1 | 0.021 | 2.42 | 0.1409 |
| *X_2_X_3_* | 0.32 | 1 | 0.32 | 36.69 | < 0.0001^**^ |
| *X_2_X_4_* | 0.66 | 1 | 0.66 | 76.35 | < 0.0001^**^ |
| *X_3_X_4_* | 0.13 | 1 | 0.13 | 15.31 | 0.0014^**^ |
| *X_1_^2^* | 1.66 | 1 | 1.66 | 191.19 | < 0.0001^**^ |
| *X_2_^2^* | 1.42 | 1 | 1.42 | 163.18 | < 0.0001^**^ |
| *X_3_^2^* | 0.64 | 1 | 0.64 | 73.32 | < 0.0001^**^ |
| *X_4_^2^* | 0.94 | 1 | 0.94 | 107.91 | < 0.0001^**^ |
| Residual | 0.13 | 15 | 0.0087 |  |  |
| Lack of Fit | 0.11 | 10 | 0.011 | 3.48 | 0.0907 |
| Pure error | 0.016 | 5 | 0.0033 |  |  |
| Total dispersion | 6.96 | 29 |  |  |  |
| *R*^2^ | 0.9812 | Adj-*R*^2^ | 0.9637 | Pred-*R*^2^ | 0.9012 |
| Std. Dev | 0.093 | Mean | 11.9600 | C. V. % | 0.7800 |
